# Compound Fracture of the Skull

**Published:** 1844-05-04

**Authors:** 


COMPOUND FRACTURE OF THE SKULL

A boy twelve years of age, was admited into Jervis-street Hospital under Dr. Banon, having been previously knocked down and stunned by a kick from a horse on the left side of the head. On examination there was found a compound and comminuted fracture, engaging the upper and anterior part of the left parietal bone, near its anterior and superior angle. It was a flap wound, patent and crescentic, with its convexity towards the temporal bone. The posterior angle of the fracture was much indented, having, probably, been opposed to the cock of the shoe, and the bone was forced through the dura mater upon the brain, some of which escaped through the wound. The boy was conscious, and apparently in possession of all his faculties except speech. The next day some symptoms of facial paralysis existing, Dr. Banon exhibited mercury with the view of causing salivation, which was produced in a few days with advantage. Suppuration took place by the eighth day, causing, by its pressure on the brain, severe cerebral symptoms, until it was freely discharged by the wound. This state recurred once afterwards from the same cause, and was relieved in a similar manner. By two months after the accident, the boy was perfectly recovered, some slight exfoliation of bone having taken place.—*Ibid.*

